# The Impact of the Lunar Cycle and Season on Small Mammal Communities Near a Large Metropolitan Area

**DOI:** 10.1002/ece3.71237

**Published:** 2025-05-21

**Authors:** Tasha Oosthuizen, Maria K. Oosthuizen, Neville Pillay

**Affiliations:** ^1^ School of Animal, Plant and Environmental Sciences, University of Witwatersrand Johannesburg South Africa; ^2^ Department of Zoology and Entomology University of Pretoria Hatfield South Africa; ^3^ Mammal Research Institute, University of Pretoria Hatfield South Africa

**Keywords:** abundance, demography, lunar cycle, small mammals, South Africa

## Abstract

Small mammals are prey to a range of predators and consequently use environmental cues, such as light, to assess the level of predation risk in their environment. The lunar cycle significantly influences the fear landscape for prey species because their risk level varies with visibility. We conducted small mammal surveys in two communities in close proximity to each other on a peri‐urban site in South Africa. We investigated the abundance, composition and diversity of these communities across the lunar cycle and seasons. As predicted, we trapped the highest number of individuals and species on new moon nights, which have low light levels and thus indicate a reduced perceived predation risk. Winter showed a higher abundance compared to the other seasons, which could be explained by a decline in naturally available resources in winter. We captured more individuals and species on one site compared to the other site, largely because of the different microhabitats in the two sites; one site had a lower density of trees and more bare soil areas. Our study provides the first evidence of the impact the lunar cycle has on small mammal communities in southern Africa. Based on our findings of decreased activity of small mammals under increased lunar visibility, we hypothesise that small mammal communities in southern Africa will be negatively affected by the increased occurrence of artificial light at night, which could have wider ecosystem impacts.

## Graphical abstract

Our study is one of the first studies to investigate the influences of the lunar cycle on African small mammals. Their abundance and composition decreased during a full moon night, which suggests a strong and universal response to increased illumination. There was a seasonal influence on the small mammal community, as we expected, and it seems like it was mostly related to a range of biotic and abiotic factors that could have acted in concert with each other.

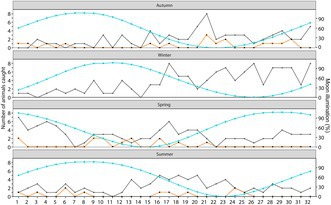

## 1 Introduction

Rodents are prey species and often need to balance the benefits of foraging with the potential risks of predation (Lima and Dill 1990; Mandelik et al. 2003). Most rodents are nocturnal (Hawkins and Golledge 2018) and can use the moonlight to aid in their movement under low light levels. However, the intensity of the natural light changes depend on the moon phase (Weaver 2011). A full lunar cycle is 29.5 days with the light intensity of a new moon being approximately 0.0001 Lux, whereas a full moon can reach intensities of up to 2 Lux (Weaver 2011; Penteriani et al. 2013). The intensity of the moonlight affecting animals also depends on the vegetation density and complexity, which lessens visibility under bright moonlight (Guiden and Orrock 2019).

Predation risk increases during brighter nights because prey are easier to locate, which prompts behavioural changes such as increased alertness and decreased activity in prey species (Prugh and Golden 2014; Russart and Nelson 2018). Rodents suppress their activity under brighter moon nights as an antipredator strategy (Prugh and Golden 2014). On moonlit nights, prey animals typically avoid open areas due to the elevated predation risk (Mandelik et al. 2003). Several examples illustrate this behaviour: fewer wood mice ( *Apodemus sylvaticus* ) were trapped during full moon nights compared to new moon nights (Perea et al. 2011). The higher illumination from a full moon improved the foraging efficiency of short‐eared owls ( *Asio flammeus* ), whereas their prey species, deer mice ( *Peromyscus maniculatus* ), reduced their activity and feeding (Clarke 1983). Nocturnal common spiny mice ( *Acomys cahirinus* ), which foraged in an open habitat, were significantly influenced by moonlight, visiting fewer artificial food trays to limit detection under moonlit nights (Mandelik et al. 2003). Allenby's gerbils (*Gerbillus andersoni allenbyi*) were more vigilant during the brightest full moon phase, followed by the waning moon, waxing moon and lastly the new moon, and they ceased foraging sooner during the waxing moon, followed by the full, new and waning moon (Kotler et al. 2010). During the brighter part of the lunar cycle (full and waxing moon), these gerbils also increased vigilance and reduced foraging, resulting in a poorer body condition, but as the cycle progressed and starvation increased, the gerbils spent more time foraging despite exposure and potential predation risk (Kotler et al. 2010). Prey species, such as rabbits ( *Oryctolagus cuniculus* ) also travel longer distances and use simpler movement patterns as their main predator avoidance strategy during a new moon compared to full moon nights (Penteriani et al. 2013). Prey animals thus modify their behaviour to minimise the risk of exposure and detection by predators during nights with high visibility.

Within a small mammal community, there are a multitude of natural factors that can affect the population dynamics of individual species. Biotic factors, such as intra‐ and inter‐specific competitors and predators, and abiotic factors, such as climatic factors and the lunar cycle, act in concert to create the spatial and temporal niche of an animal (Pratas‐Santiago et al. 2017). The combination of these factors creates a complex system and teasing apart the components of these systems is necessary to understand the wider processes and dependencies in an ecosystem (Radchuk et al. 2016). For example, the quantity of available food is dependent on rainfall (Shilereyo et al. 2023). Moreover, the quantity of seasonal rainfall influences the density of vegetation cover, which could impact how small mammals move on the ground and how they perceive moonlight (Guiden and Orrock 2019). Foraging success is also affected by the lunar cycle. Depending on levels of satiety, prey species could be willing to take risks if they need to forage to meet their energy needs or reduce foraging if they are not energetically compromised (Bedoya‐Perez et al. 2013). The concept of landscape of fear has been used to understand the use and timing of different activities to minimise risk while considering the cumulative effects of various environmental factors (van der Merwe and Brown 2008; Gaynor et al. 2019).

We investigated how the abundance (number of animals) and composition (number of species) of small mammals differ over the lunar cycle and seasonally. Small mammal trapping surveys were conducted in two areas at a peri‐urban field site, outside Johannesburg, South Africa. We predicted that (1) because the percentage moon illumination is associated with the landscape of fear (i.e., perceived levels of predation risk), greater moon illumination (days around a full moon) would result in lower trapping success, whereas reduced moon illumination (days around a new moon) would show a higher trapping success, regardless of the trapping sites. Additionally, because small mammal populations fluctuate seasonally based on the available resources, we also predicted that (2) the small mammal composition and abundance would be more diverse and higher, respectively, in autumn and winter when the naturally occurring resources are lower and baited traps become an available food source. In contrast, we expected a less diverse composition and lower abundance during spring and summer when there are sufficient available resources.

## 2 Materials and Methods

### 2.1 Trapping Sites

The research was conducted on the Cradle Nature Reserve (−25.9214, 27.8503) located within the Magaliesberg Biosphere in the Gauteng Province of South Africa. The site is located approximately 30 km from the centre of Johannesburg, the largest metropolitan area in southern Africa. This area consists of both savanna and grassland biomes with some woody vegetation and herbaceous grasses (Mucina and Rutherford 2006; Ramahlo et al. 2022). Two trapping sites were randomly identified and were designated as Site 1 and Site 2 (Figure S1). The two sites were approximately 1.7 km apart and had a similar slope aspect, facing south and to a comparable gradient (Pinto 2024). Site 1 had a more complex vegetation structure: The vegetation was generally taller and provided greater ground cover compared to Site 2 (Pinto 2024). In addition, there were more trees on Site 1 with less exposed soil compared to Site 2. Bronze love grass (*Eragrostis heteromera*) was the dominant grass species during both summer and winter on the Cradle Nature Reserve (Pinto 2024). However, the vegetation of the site has not been comprehensively documented. The study site is located in an area with a hot, wet summer and cold, dry winter. The seasons were allocated as follows: summer—December to February, autumn—March to May, winter—June to August and spring—September to November.

### 2.2 Animal Trapping and Measurements

We trapped small mammals in four seasons within 1 year (2022/2023; Table S1), using 75 PVC live animal traps per site (7.5 × 7.5 × 30 cm) laid out on a permanent grid (traps separated by 10 m in five rows of 15 traps each). These PVC traps are an alternative to Sherman live traps, but it differs in the way the door closes; an animal steps on a metal plate, and the door then falls shut from above rather than from below (Figure S2). Traps were covered with loose vegetation to aid in insulation and mimic a more natural appearance. When possible, we placed traps close to trees or large rocks, but when those were not present in the immediate vicinity, we placed them in bushes, either facing outward or, in the case of high‐use rodent highways, we placed them facing inward toward the runways. The positioning of the traps was similar on both sites. The traps were baited with a mixture of sunflower seeds, sunflower oil, rolled oats, granola and salt. Traps were opened at approximately 17h00 every day and checked the following morning at sunrise (between 05h00 and 06h30 min depending on the season). Traps were closed during the day because we were interested in the activity of nocturnal rodents to assess the impact of the lunar cycle on this guild.

Trapping lasted for a total of 32 continuous days per season (Table S1) to account for the varying illumination levels throughout a full lunar cycle. During winter, cotton wool was placed in the traps to provide some warmth for the trapped animals on cold nights. When we confirmed that a trap contained a small mammal, we emptied the contents of the trap into a transparent plastic Ziploc freezer bag to identify the animal to species level where possible. Three cryptic species that were not morphologically identifiable were later identified through mtDNA cyt *b* sequencing using tissue from trapped individuals, obtained by cutting a small piece of the animal's external ear. We weighed the animal using a hanging scale (1 g precision; Pesola, Switzerland). We sexed each animal using its anogenital distance; larger in males than females. Each animal was fitted with a pair of unique ear tags (National Band & Tag Company, USA) to identify re‐captured individuals. The individual was then released at the site of its capture. A trapping permit was approved by the Gauteng Department of Agriculture and Rural Development (CPF6‐0231) and the University of Witwatersrand Animal Research Ethics Committee provided ethical clearance for this study (2021/08/09B).

The moon illumination data (percentage of the moon lit up per day) were downloaded from the Time and Date website (https://www.timeanddate.com/moon/south‐africa/johannesburg?month=3&year=2023, accessed April 2023) and the website calculated the data at lunar noon, and it accounted for refraction. In addition, weather data from the nearest weather station at Lanseria, Johannesburg (located approximately 10 km from the trapping sites) was accessed through the VisualCrossing website (https://www.visualcrossing.com/weather/weather‐data‐services#, accessed April 2023). The downloaded weather variables included the minimum temperature, wind speed, cloud cover, humidity and precipitation on a trapping day.

### 2.3 Data Analyses

In winter, Site 2 burned down completely after only 10 days of trapping because of a runaway fire. Thus, winter trapping could not be completed and was not included in the analysis for Site 2. Since Site 1 was not in close proximity to the fire, we continued trapping there during winter. Species richness was calculated for site and season, using the count of species per site and per season. All data analyses were done using R software (R core team, 2022, v4.2.1). We calculated the diversity of small mammals by site and season using the Shannon and Simpson diversity indices and Pielou's evenness index in the ‘vegan’ package (Oksanen et al. 2022). The Shannon and Simpson indices indicate the concentration of individuals per species in the community (Manyonyi et al. 2020). Pielou's evenness index compares how abundant each species is in the community (Jost 2010). We included different diversity indices because it aids in a comprehensive understanding of complex interactions in a community (Morris et al. 2014) when there are multiple contributing factors in the results. It is common practice in small mammal community studies, for example, Avenant 2011; Manyonyi et al. 2020 and Ramahlo et al. 2022. To analyse whether the indices differed between sites and seasons, we ran a Kruskal–Wallis test for the Simpson index because the data were non‐normally distributed (Shapiro–Wilk test: *p* < 0.05) and *t*‐tests for the Shannon and Pielou indices, which were normally distributed (Shapiro–Wilk test: *p* > 0.05). In order to assess the homogeneity of populations across sites and seasons, we used the *betadisper* function to generate a Bray–Curtis dissimilarity score (the difference in the species composition between groups), using means per site per season (Oksanen et al. 2022).

We used linear models to analyse which factors influenced the abundance and composition of the populations. The actual data of the response variables, abundance of animals caught (including new and recaptured animals) and the composition (the number of different species caught per day) were considered daily to coincide with the changes in moon illumination and cloud cover. These variables were non‐normally distributed (*p* < 0.05; Shapiro–Wilk test) and were analysed using generalised linear models (GLMs), with a Poisson distribution and log link function. Predictor variables included the site, season, moon illumination (percentage), cloud cover (percentage), the interaction between site and season, the interaction between moon illumination and cloud cover, and a principal component made up of weather variables. The weather variables included in the principal component analysis (PCA) included the minimum temperature, wind speed, humidity and precipitation. The PCA was constructed using the ‘FactoMineR’ (Husson et al. 2023) and ‘factoextra’ (Kassambara and Mundt 2022) packages in R. Four different principal components (PCs) were extracted (Table S3) and the PC explaining the highest percentage of the variance was considered in the GLMs.

To obtain the most parsimonious model per response variable, we used the *drop1* function to remove non‐significant variables in a stepwise manner. All model versions were then compared using the ‘MuMIn’ package (Barton 2023), and the model with the highest weight and lowest AICc was used for all reported results per response variable (Table 1). Post hoc comparisons were completed for all significant categorical variables using the ‘emmeans’ package (Lenth et al. 2020). If the continuous variables had a significant influence on the response variables, it was further analysed using Spearman rank correlations. All tests were two‐tailed, and model significance was set at 0.05. Site as a predictor variable could introduce pseudo‐replication. Thus, we compared our most parsimonious model per response variable (Table 1) with two alternative models. The first alternative model included site as only a random variable, not a predictor variable (Table S2). The second alternative model included site as both a predictor variable and a random variable (Table S2). A comparison of all three models using the AICc and weight values showed that the original model with site as a predictor variable was the most parsimonious for both response variables and thus was retained in our analysis (Table S2).

**TABLE 1:** The models considered for the number of animals and species caught.

Model	Variables	df	AICc	Delta	Weight
Animal abundance (i.e., number of animals caught)					
1	Season, site, % moon illumination, % cloud cover, season*site, % moon illumination*cloud cover, PC1	12	673.40	3.76	0.083
1.1	Season, site, % moon illumination, % cloud cover, season*site, % moon illumination*cloud cover	11	671.20	1.54	0.252
**1.2**	Season, site, % moon illumination, % cloud cover, % moon illumination*cloud cover	**8**	**669.60**	**0.00**	**0.542**
1.3	Season, site, % moon illumination, % cloud cover	7	675.20	5.55	0.034
1.4	Season, site, % moon illumination	6	673.40	3.73	0.084
1.5	Site, % moon illumination	3	678.80	9.19	0.005
Species composition (i.e., number of species caught)					
2	Season, site, % moon illumination, % cloud cover, season*site, % moon illumination*cloud cover, PC1	12	559.60	6.23	0.016
2.1	Season, site, % moon illumination, % cloud cover, season*site, % moon illumination*cloud cover	11	557.40	4.01	0.047
2.2	Season, site, % moon illumination, % cloud cover, season*site	10	555.60	2.13	0.120
**2.3**	Season, Site, % Moon illumination, Season*Site	**9**	**553.4**	**0.00**	**0.350**
2.4	Season, site, % moon illumination	6	554.30	0.92	0.221
2.5	Site, % moon illumination	3	554.10	0.71	0.246

*Note:* Models 1 and 2 are the saturated models for the two response variables. The number of variables was reduced in a stepwise manner, using the *drop1* function. The parsimonious models are *bolded* and were chosen as the models with the lowest AICc and highest weight values.

## 3 Results

The models for the statistical analyses considered all predictor variables and interactions between categorical and continuous variables separately. The final models used depended on which variables were retained in the analyses (Table 1).

### 3.1 Trapping Success

**FIGURE 1:**
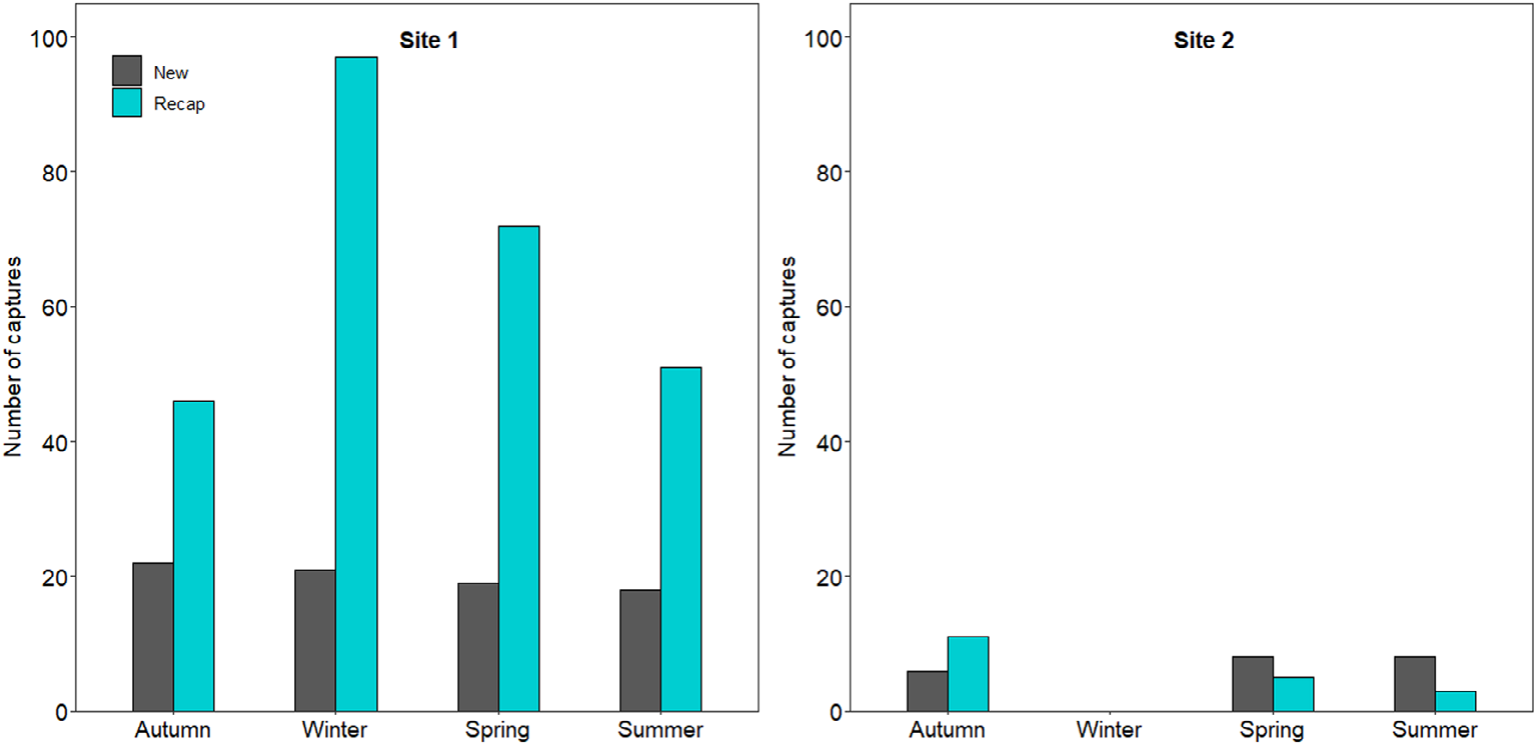
The number of small mammals captured during each season on each of the two trapping sites at the Cradle Nature Reserve, South Africa. There are no data displayed for winter on Site 2 because it burned down during the trapping period.

In the 128 trapping days (19,200 trap nights), a total of 396 small mammals were caught, of which 72.2% were recaptured individuals (Figure 1). The highest number of new captures was during autumn on Site 1 and during spring and summer on Site 2 (Table 2 and Figure 1). The highest number of recaptured animals occurred during winter on Site 1 and during autumn on Site 2 (Table 2 and Figure 1).

**TABLE 2:** The abundance of small mammals and species richness by season on Site 1 and Site 2 at the Cradle Nature Reserve, South Africa.

	Autumn		Winter		Spring		Summer	
	Site 1	Site 2	Site 1	Site 2	Site 1	Site 2	Site 1	Site 2
Chestnut climbing mouse (*Dendromys mystacalis*)	0	0	1	NA	0	0	0	0
Bushveld gerbil (*Gerbillicus leucogaster*)	3	2	1	NA	2	4	1	5
Southern multimammate mouse ( *Mastomys coucha* )	11	0	11	NA	7	4	11	0
Namaqua rock mouse ( *Micaelamys namaquensis* )	0	2	1	NA	0	0	0	2
Pygmy mouse ( *Mus minutoides* )	0	0	4	NA	8	0	0	0
Angoni vlei rat ( *Otomys angoniensis* )	7	1	1	NA	2	0	3	1
Four‐striped grass mouse (*Rhabdomys dilectus chakae*)	0	0	0	NA	0	0	3	0
Musk shrew (*Crocidura* spp.)	1	1	2	NA	0	0	0	0
Total new captured animals	22	6	21	NA	19	8	18	8
Total recaptured animals	46	11	97	NA	72	5	51	3
Total captured animals	68	17	118	NA	91	13	69	11
Sex ratio (males/females)	1.00	0.89	7.14	NA	0.66	3.33	1.96	0.83
Species richness (*N*)	4	4	7	NA	4	2	4	3

*Note:* *Mastomys coucha* , *Otomys angoniensis* and *R. d. chakae* were identified genetically. The NAs under Site 2 in winter indicate no trapping because the site burned down.

We caught significantly more individual small mammals on Site 1 compared to Site 2 (*χ* ^2^ = 188.54, df = 1, *p* < 0.001, Figure 1). Season influenced the number of small mammals caught (*χ* ^2^ = 11.72, df = 3, *p* = 0.008, Figure 1). Post hoc comparisons showed that the number of animals caught during winter was higher than both autumn and summer (*p* ≤ 0.036). None of the other comparisons were significant (*p* ≥ 0.259).

Several species were caught on both sites, including southern multimammate mice ( *Mastomys coucha* ), bushveld gerbils (*Gerbillicus leucogaster*), Angoni vlei rats ( *Otomys angoniensis* ), Musk shrews (*Crocidura* spp.) and Namaqua rock mice ( *Micaelamys namaquensis* ) (Table 2). The chestnut climbing mouse (*Dendromys mystacalis*), pygmy mice ( *Mus minutoides* ) and four‐striped grass mice (*Rhabdomys dilectus chakae*) were only caught on Site 1. None of the species was unique to Site 2 (Table 2). The identity of *Otomys angoniensis* , *Rhabdomys dilectus chakae* and *Mastomys coucha* was confirmed genetically.

We caught significantly more species on Site 1 compared with Site 2 (*χ* ^2^ = 116.74, df = 1, *p* < 0.001). Season did not significantly influence the number of species caught (*χ* ^2^ = 6.05, df = 3, *p* = 0.109), nor did the interaction between site and season (*χ* ^2^ = 7.35, df = 3, *p* = 0.061).

### 3.2 Diversity Indices

The Simpson diversity index was the highest for Site 2 during autumn (Table 3). During summer, the Simpson diversity index was similar on Sites 1 and 2 (Table 3). Site 2 had the lowest diversity during spring (Table 3) and this low diversity was also supported by the Shannon index (Table 3). The Shannon index revealed the highest diversity during winter at Site 1 (Table 3). The Pielou's evenness index indicated that the evenness was high for all sites in all seasons, with the lowest during winter at Site 1 (Table 3).

**TABLE 3:** Bray–Curtis dissimilarity and diversity indices by season and site for small mammal trapping on the Cradle Nature Reserve, Gauteng, South Africa.

Bray–curtis	Autumn		Winter		Spring		Summer	
	Site 1	Site 2	Site 1	Site 2	Site 1	Site 2	Site 1	Site 2
Winter	NA	NA	—	—	—	—	—	—
Spring	0.25	0.67	0.27	1.00	—	—	—	—
Summer	0.25	0.14	0.46	0.50	0.25	0.60	—	—
Simpson index	0.63	0.72	0.67	NA	0.67	0.50	0.57	0.53
Shannon index	1.12	1.33	1.46	NA	1.21	0.69	1.06	0.90
Pielou's index	0.81	0.96	0.75	NA	0.87	1.00	0.76	0.82

The Bray–Curtis dissimilarity composite score showed that Site 1 had a similar composition of species in all seasons. The dissimilarity score indicated that Site 2 had a similar species composition in spring and autumn, but a less diverse species composition during summer and autumn. Due to the fire, we did not consider these values in winter. The Bray–Curtis dissimilarity mean was 0.29 for Site 1 and 0.59 for Site 2 (Table 3).

### 3.3 Moon Illumination and Cloud Cover

**FIGURE 2:**
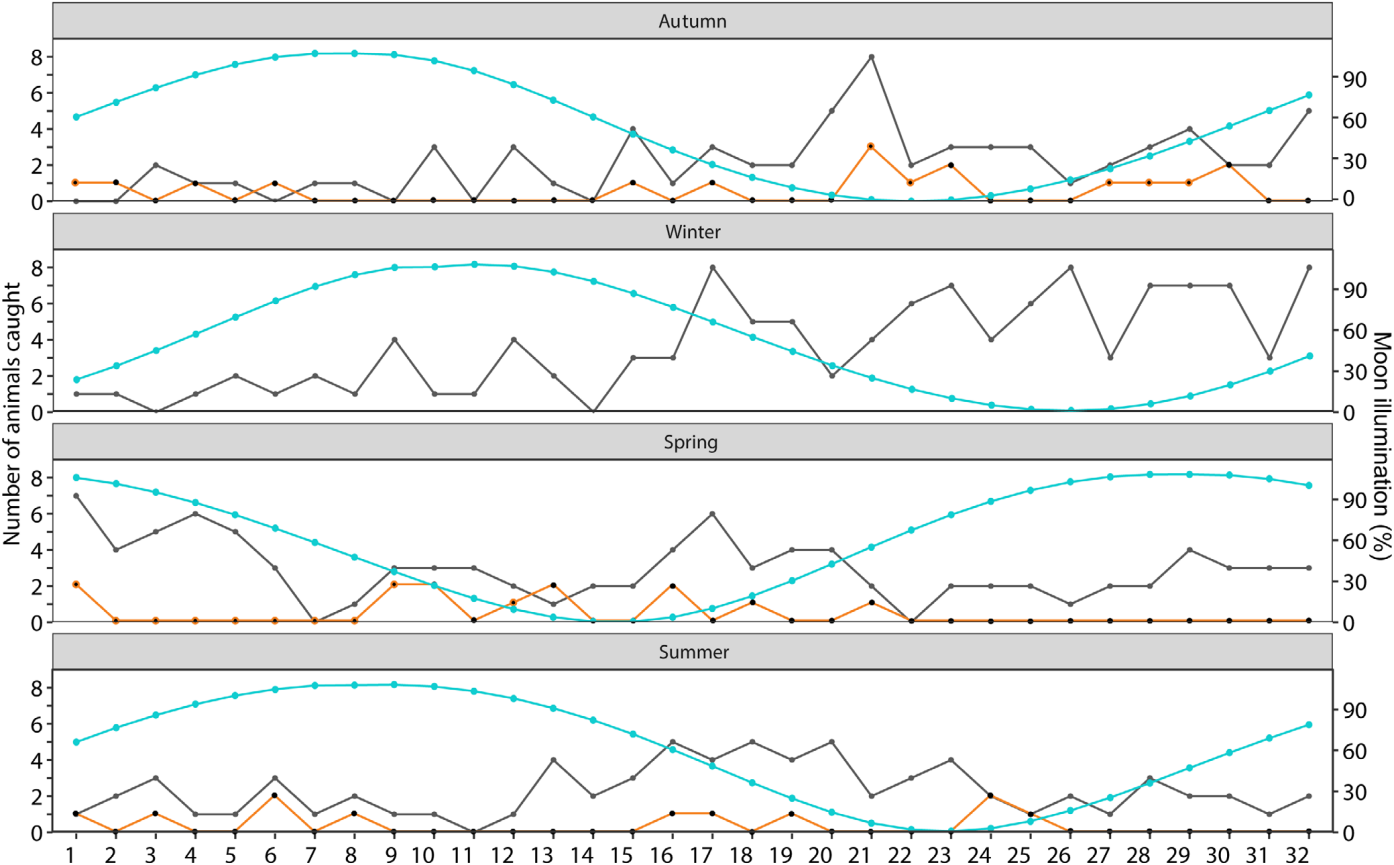
The total number of captures per day per season on Site 1 (grey line) and Site 2 (orange line) with the associated moon illumination percentage (blue line) of each day at the Cradle Nature Reserve, South Africa. No orange line is present in winter because Site 2 burned down, and no animals were caught. The percentage moon illumination data were retrieved from https://www.timeanddate.com/moon/south‐africa/johannesburg?month=3&year=2023.

Moon illumination, regardless of season and site, was a significant predictor of the number of small mammals captured (*χ* ^2^ = 25.95, df = 1, *p* < 0.001; *R* = −0.25, Figure 2). During brighter moonlight (i.e., close to the full moon), fewer animals were captured compared with nights with a new moon. The cloud cover was not a significant predictor of the number of small mammals caught (*χ* ^2^ = 0.312, df = 1, *p* = 0.576). However, the interaction between moon illumination and cloud cover was (*χ* ^2^ = 7.691, df = 1, *p* = 0.006). On days with a new moon, we caught more species (*χ* ^2^ = 11.35, df = 1, *p* < 0.001; *R* = −0.24, Figure 2).

## 4 Discussion

We investigated how the lunar cycle (moon illumination) and season influenced the small mammal abundance, species composition and diversity on two sites near Johannesburg, the largest metropolitan area in southern Africa. As predicted, we caught significantly more individuals (abundance) and species (composition) on nights of a new moon, and capture rates declined over the lunar cycle, with the lowest capture rate around a full moon. Past studies indicated that rodents decrease their overall activity with increased visibility (Penteriani et al. 2013; Pratas‐Santiago et al. 2016), as reported for wood mice ( *A. sylvaticus* ), especially in areas with limited vegetation cover (Perea et al. 2011). Since our trapping sites were geographically close together, the moon illumination was similar for the two sites. Thus, a similar pattern of low capture rates under brighter moon illumination was apparent on both sites. Yet, it is vital to note that none of these factors act in isolation, and there could be multiple factors that resulted in our findings. Available resources, the lunar cycle, weather variables, and geographic location all influence each animal simultaneously, which facilitates their activities (Falcón et al. 2020). The raw data of rodent captures appeared to be higher during the new moon in both autumn and winter and slightly lower during spring and summer and is probably linked to the availability of resources, but this relationship was not explicitly tested.

The lowest species diversity on Site 1 occurred during summer, during the rainy season with an associated increase in available natural resources. Seasonal diversity indices were the lowest on Site 2 during spring, when there was sparse vegetation following the veld fire at the end of the winter. This could have resulted in the low diversity, because only some species (such as *M. coucha* ) can tolerate the lack of resources and the increased exposure by being very alert (Oosthuizen et al. 2024). Diversity was higher for the other seasons for both sites, indicating the sites were not dominated by a single species. It is possible that during autumn and winter, when the naturally occurring resources were low, multiple species preferred the bait, resulting in a higher number of captures. However, the diversity index scores (dependent on sample size) of Site 2 were affected by the lower number of captures and should be interpreted with caution (Bashalkhanov et al. 2009). The evenness scores remained similar in all seasons and between the sites, suggesting that the small mammal communities were close to even with regard to species diversity.

The abundance of small mammals was greater in winter than autumn, spring and summer, which is applicable to Site 1 because Site 2 had a fire during winter. There are several possible explanations for this outcome. One explanation could be that the capture success was based on whether the bait was preferred over the surrounding food availability in the habitat. The small mammals on our sites usually breed during the hot and wet seasons in spring and summer (Skinner and Chimimba 2005). During these months, vegetation density is greater because of higher precipitation. The increased vegetation density is linked to increased resource abundance (Shilereyo et al. 2023), resulting in fewer captures because the small mammals could have preferred their natural food over the bait in the traps (Adler and Lambert 1997; Aplin et al. 2003). In addition to the bait potentially being more readily available than natural food, the traps could also have been appealing as shelters. In autumn, nine traps (three on the Site 2 and six on Site 1) contained nests; it appeared that the animals managed to enter and leave traps repeatedly during a single night and carried in nesting material. Similarly, during winter, there were eight such instances, all on Site 1. This did not occur during spring and summer, when the surrounding vegetation cover was better.

An alternative explanation is that the small mammals expanded their home ranges (outside of the trapping sites) in spring and summer, in search of potential mates and possibly also experienced increased mortality as a result of competitive interactions, resulting in lower capture rates (Rocha et al. 2016; Ramahlo et al. 2022). We caught fewer small mammals during spring on both sites, but Site 2 burned down in the preceding winter, and most likely contributed to the lower diversity. This would have reduced the availability of resources and increased exposure to predation risk (González et al. 2022). The exception would be the multimammate mouse, a post‐burn pioneer species (Leirs et al. 1994) that occurred in Site 2 following the burn. The multimammate mouse is skittish (Oosthuizen et al. 2024) and this is possibly linked to its success in navigating the open habitat and avoiding predator detection. Another contributing factor could be weather variables, but the PCA analyses contributed little to our model selection and were excluded from the analyses. Cloud cover was included in the analysis because of the link between moonlight and visibility, yet it was not significant when retained in the animal abundance model. Nonetheless, this does not indicate that cloud cover had no influence, because it could still impact visibility and predation risk, by either concealing a full moon or reflecting ALAN from the city (Kyba et al. 2011) at a finer movement scale and could be an important factor to consider in future studies.

Site 1 had a higher abundance and more complex composition of small mammals compared to Site 2. This could potentially be explained by microhabitat differences between the sites, which can influence food availability and predation risk (Longland and Price 1991; Penteriani et al. 2013; Mori et al. 2020). Site 1 appeared to have a structurally more complex habitat with regard to the range of vegetation cover and height than Site 2 (Pinto 2024), which could indicate greater varieties of microhabitat compared to the less complex Site 2. Nonetheless, there could be other factors influencing this difference, such as the density and presence of predators (data not collected in our study).

## 5 Conclusions

We present one of the first studies to investigate the influence of the lunar cycle on African small mammals. Their abundance and composition decreased during full moon nights, which suggests a strong and universal response to increased illumination. There was a seasonal influence on the small mammal community, as we expected, and it seems like it was mostly related to a range of biotic and abiotic factors that could have acted in concert with each other. Our results suggest that small mammal communities in the southern hemisphere will face similar consequences to those from the northern hemisphere with decreased activity under increased artificial light at night (ALAN). Our study sites were located in peri‐urban Greater Johannesburg, a rapidly expanding metropolitan city. Our findings suggest that the small mammal communities on our study site could experience the increasing demands of constant ALAN in addition to natural variation in lunar cycles. These are likely to lead to an increasing risk of predation and trade‐offs with foraging. How these changes impact the broader ecosystem will be a fruitful avenue of future research.

## Author Contributions

**Tasha Oosthuizen:** conceptualization (equal), formal analysis (lead), investigation (equal), methodology (equal), project administration (lead), visualization (equal), writing – original draft (lead). **Maria K. Oosthuizen:** conceptualization (equal), investigation (equal), methodology (equal), supervision (equal), visualization (equal), writing – review and editing (equal). **Neville Pillay:** conceptualization (equal), funding acquisition (lead), methodology (equal), resources (lead), supervision (equal), writing – review and editing (equal).

## Conflicts of Interest

The authors declare no conflicts of interest.

## Supporting information


Data S1.

## Data Availability

Data used in this study can be found at https://figshare.com/s/d99a1c7ce7ddbcce1f97.
