# Supplementary material for: The Impact of the Lunar Cycle and Season on Small Mammal Communities Near a Large Metropolitan Area
Source: Ecol Evol. 2025 May 21;15(5):e71237. doi: 10.1002/ece3.71237 (PMC12094881; doi:10.1002/ece3.71237)
Supplement: Supplementary file 1 — Data S1. [file ECE3-15-e71237-s001.docx]

**The impact of the lunar cycle and season on small mammal communities near a large metropolitan area**

Tasha Oosthuizen^a^, Maria K Oosthuizen^a,b,c^ and Neville Pillay^a^

^a^School of Animal, Plant and Environmental Sciences, University of Witwatersrand, Private Bag 3, WITS, Johannesburg, 2050, South Africa

^b^Department of Zoology and Entomology, University of Pretoria, Private Bag X20, Hatfield, 0028, South Africa

^c^Mammal Research Institute, University of Pretoria, Hatfield, 0028, South Africa

E-mail addresses: [Tasha.Oosthuizen@wits.ac.za](mailto:Tasha.Oosthuizen@wits.ac.za) (T Oosthuizen), [moosthuizen@zoology.up.ac.za](mailto:moosthuizen@zoology.up.ac.za) (MK Oosthuizen), [Neville.Pillay@wits.ac.za](mailto:Neville.Pillay@wits.ac.za) (N Pillay).

**Corresponding author:** T Oosthuizen

[Tasha.Oosthuizen@wits.ac.za](mailto:Tasha.Oosthuizen@wits.ac.za)

**Supplementary Tables**

Supplementary Table 1. The seasonal sampling period dates for Sites 1 and 2 on the Cradle Nature Reserve.

| **Seasons** | **Start date** | **End date** |
| --- | --- | --- |
| Autumm | 10/05/2022 | 10/06/2022 |
| Winter | 03/08/2022 | 03/09/2022 |
| Spring | 11/11/2022 | 12/12/2022 |
| Summer | 30/01/2023 | 02/03/2023 |

Supplementary table 2. The models considered for the number of animals and species caught. Models 1 and 2 are the saturated models for the two response variables, models 1a and 2a has Site as the random variable instead of a predictor variable and 1b and 2b contains Site as both a predictor and random variable. The parsimonious models are bolded and were chosen as the models with the lowest AICc and highest weight values.

| **Animal abundance (i.e., number of animals caught)** | | | | | |
| --- | --- | --- | --- | --- | --- |
| **Model** | **Variables** | **df** | **logLik** | **AICc** | **Weight** |
| **1** | **Season, Site, % Moon illumination, % Cloud cover, % Moon illumination*Cloud cover**  **(NO random variable)** | **8** | **-326.50** | **669.6** | **0.745** |
| 1a | Season, % Moon illumination, % Cloud cover, % Moon illumination*Cloud cover  (Site = random variable) | 8 | -332.15 | 680.9 | 0.003 |
| 1b | Season, Site, % Moon illumination, % Cloud cover, % Moon illumination*Cloud cover  (Site = random variable) | 9 | -326.50 | 671.8 | 0.253 |
| **Species composition (i.e., number of species caught)** | | | | | |
| **2** | **Season, Site, % Moon illumination, Site*Season**  **(NO random variable)** | **8** | **-326.50** | **669.6** | **0.745** |
| 2a | Season, % Moon illumination, Site*Season  (Site = random variable) | 8 | -332.15 | 680.9 | 0.003 |
| 2b | Season, Site, % Moon illumination, Site*Season  (Site = random variable) | 9 | -326.50 | 671.8 | 0.253 |

Supplementary Table 3. The contributions of each weather variable to four separate principal components (PCs). The eigenvalue, variance percentage and cumulative variance percentage of each PC is included, with the most representative PC (explaining the highest percentage of the variance and an eigenvalue larger than 1) indicated with an asterisk.

|  | **PC1*** | **PC2** | **PC3** | **PC4** |
| --- | --- | --- | --- | --- |
| Minimum temperature | 0.580 | -0.031 | 0.581 | -0.570 |
| Wind speed | 0.157 | 0.950 | 0.127 | 0.237 |
| Humidity | 0.613 | -0.295 | 0.086 | 0.728 |
| Precipitation | 0.513 | 0.096 | -0.799 | -0.299 |
| **Eigenvalue** | 1.923 | 1.021 | 0.711 | 0.346 |
| **Variance %** | 48.077 | 25.514 | 17.763 | 8.645 |
| **Cumulative variance %** | 48.077 | 73.592 | 91.355 | 100 |

**Supplementary Figures**

**
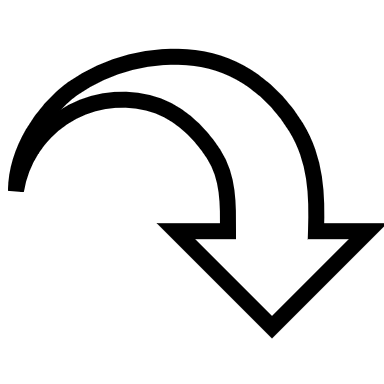

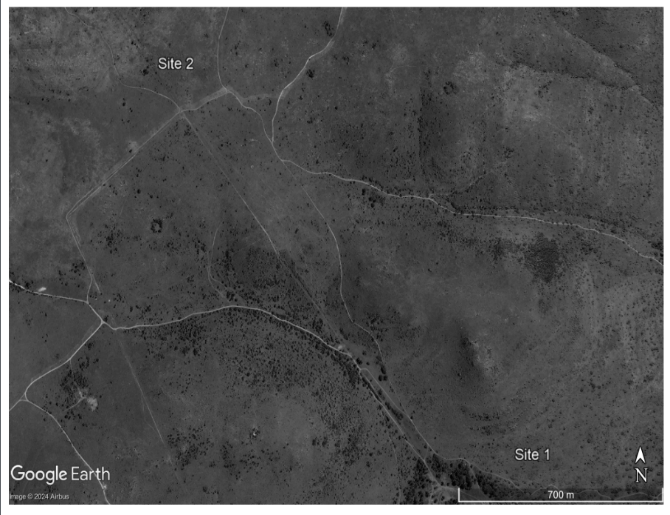
**
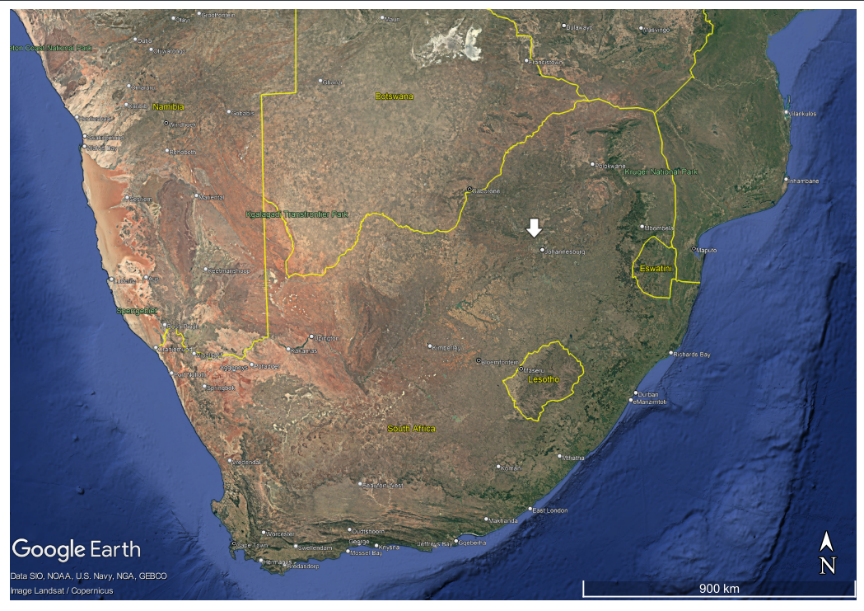


Supplementary Figure 1. The locations of Site 1 and Site 2 on the Cradle Nature Reserve property. The Site 1 faced Johannesburg and Site 2 faced the opposite direction.


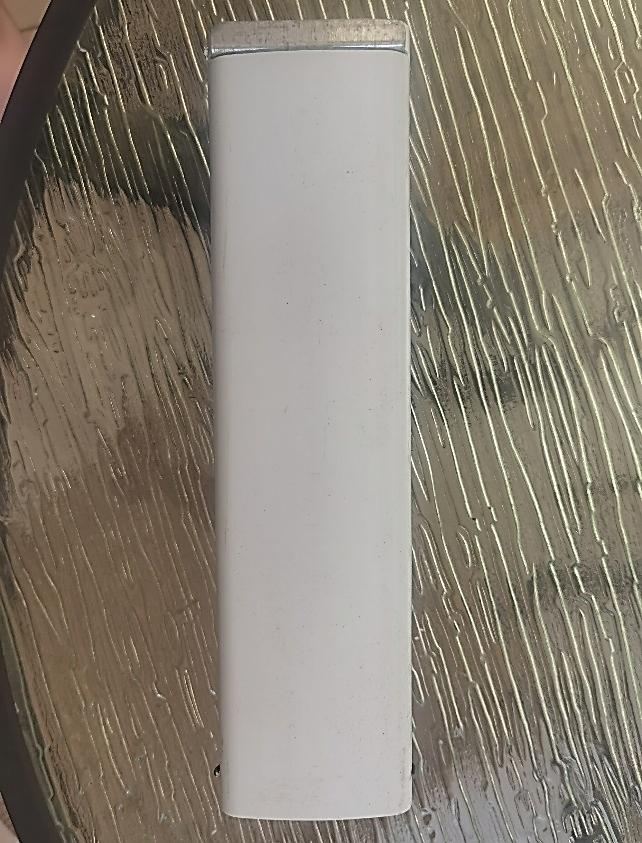

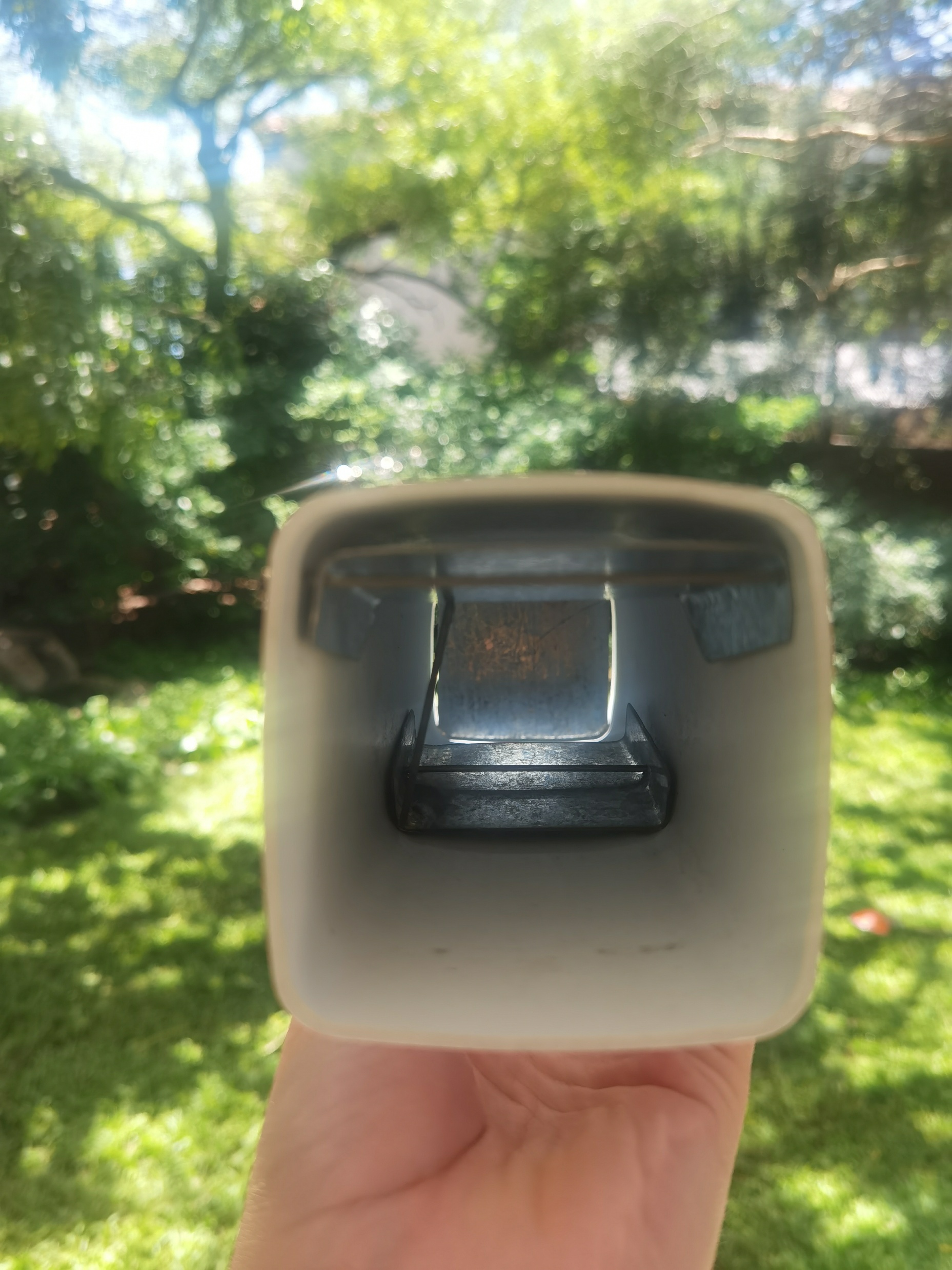


Supplementary Figure 2: Pictures of the PVC live traps used to capture small mammals. Left – front view of the trap, showing the door at the top of the trap that is kept open by a little steel arm on the lefthand side and the metal plate at the bottom of the trap that triggers the door. Right – top view of a trap.
